# Seeing the Big Picture: Size Perception Is More Context Sensitive in the Presence of Others

**DOI:** 10.1371/journal.pone.0141992

**Published:** 2015-11-12

**Authors:** Teresa Garcia-Marques, Alexandre Fernandes, Marília Prada, Ricardo Fonseca, Sara Hagá

**Affiliations:** 1 Department of Social and Organizational Psychology, ISPA- Instituto Universitário, Lisbon, Portugal; 2 William James Center of Research, ISPA-IU, Lisbon, Portugal; 3 Department of Social and Organizational Psychology, ISCTE-Instituto Universitário de Lisboa, Lisbon, Portugal; 4 Centro de Investigação e Intervenção Social-Instituto Universitário de Lisboa (Cis-IUL), Lisbon, Portugal; 5 Faculdade de Psicologia da Universidade de Lisboa, Lisbon, Portugal; Goldsmiths, University of London, UK, UNITED KINGDOM

## Abstract

This paper tests the hypothesis that social presence influences size perception by increasing context sensitivity. Consistent with Allport’s prediction, we expected to find greater context sensitivity in participants who perform a visual task in the presence of other people (i.e., in co-action) than in participants who perform the task in isolation. Supporting this hypothesis, participants performing an Ebbinghaus illusion-based task in co-action showed greater size illusions than those performing the task in isolation. Specifically, participants in a social context had greater difficulty perceiving the correct size of a target circle and ignoring its surroundings. Analyses of delta plot functions suggest a mechanism of interference monitoring, since that when individuals take longer to respond, they are better able to ignore the surrounding circles. However, this type of monitoring interference was not moderated by social presence. We discuss how this lack of moderation might be the reason why the impact of social presence on context sensitivity is able to be detected in tasks such as the Ebbinghaus illusion.

## Introduction

Allport [1] was the first to note that social presence increases context sensitivity. When performing a free-association task, individuals in the presence of other people (i.e., in co-action) exhibited more context-related responses than individuals in isolation. In his own words, social presence takes the individual “out of himself and directs his ideas toward outside objects” (pp. 167–168). Recently, Fonseca and Garcia-Marques [2], using a task that measures sensitivity to contextual information–framed-line test [3], showed further evidence of this effect. The authors asked participants to reproduce a previously observed line identically (absolute task) or proportionally (relative task) in a new surrounding frame. Participants in a social presence condition performed more accurately than those in an isolation condition when contextual information was considered (relative task).

Those results suggest that social presence is likely to modulate illusions of size perception promoted by contextual information, such as the effects usually found using an Ebbinghaus illusion experimental paradigm [4]. If such modulation exists, the increased context sensitivity in the presence of others should lead to an increase of this type of illusion in a co-action condition relatively to an isolation condition. However, social presence has been shown to increase individuals’ resistance to irrelevant interferences, too. For example, participants in Stroop-like tasks show less interference when placed in the presence of others than when in isolation [5]. Thus, if the Ebbinghaus illusion task is susceptible to the same type of monitoring mechanisms as the Stroop tasks, we may not be able to detect a social presence-related increase in context sensitivity. In that case, participants in the presence of others would demonstrate weaker size illusions than participants in an isolation condition because they would be better at controlling contextual influences.

In sum, social presence can lead to one of three results in an Ebbinghaus illusion task, through the differential operation of two mechanisms, namely enhanced context sensitivity and enhanced monitoring: (1) an increase in the Ebbinghaus illusion through an effect of social presence on context sensitivity and thus on local/global perception (i.e., similar to what is observed in the framed-line test); (2) a decrease in the Ebbinghaus illusion through an effect of social presence on interference monitoring (i.e., similar to what is observed in the Stroop task); or (3) neither an increase nor decrease in the Ebbinghaus illusion, if the two mechanisms fully cancel each other out. An analysis of the specific features of the Ebbinghaus illusion task and of how they differ from the features of a Stroop-like task may help us predict which one of these hypotheses is most likely.

### Ebbinghaus illusion task

The Ebbinghaus illusion task assesses how individuals’ size perception is sensitive to contextual features [4,6]. This forced-choice task that requires participants to select the larger of two circles presented side by side of the screen. These circles are surrounded by other circles that provide a context that can either support (facilitate) or oppose (inhibit) accurate discrimination. Facilitation trials allow participants to respond correctly either by attending to the target stimuli, to their context, or both (e.g., when a large target circle surrounded by large context circles is next to a small target circle surrounded by small context circles). Instead, in inhibition trials, participants are required to inhibit the response offered by the context (which would bias the response; e.g., a large circle surrounded by small circles) and to focus only on the difference between the sizes of both target circles.

In tasks that require inhibition of the interference exerted by the context, accurate performance may occur by the operation of, at least, one of two different mechanisms (for a review, see [7]). One mechanism occurs earlier in the processing phase—early attention selection mechanism–and controls reflexive processing by suppressing the activation of the undesirable influence. The other mechanism is a late selection mechanism in which the produced responses are inhibited (i.e., an activation plus suppression mechanism associated with executive function control).

The Ebbinghaus illusion task and the Stroop task rely differently on these two mechanisms. In contrast to what occurs in a Stroop task [6,8], the interference of the context in the Ebbinghaus illusion task is not associated with a delay of the correct responses. In the Ebbinghaus illusion task the interference modulates the actual perception of the stimulus size [9]. Being perceptual, the illusion is quickly established and its avoidance is mainly dependent upon earlier attentional mechanisms [10]. An initial focus of attention on the relevant stimuli is what increases accuracy, by decreasing perceptive illusions [11]. Once a perception is formed, it is unlikely changed, being immune to subsequent attentional processes. In other words, the Ebbinghaus illusions are expected to be immune to the reflective processing that aims to suppress undesirable influences [12]. In the Stroop task, an automatic response (e.g., seeing a color) suffers the interference of another automatic response (e.g., reading a color name). This type of interference takes time to be implemented, such that it is minimal for faster responses and increases as responses slow down. The inhibitory mechanisms operate, if at all, when interference is higher, in the later moments of the process, preventing incorrect responses [12]. Hence, Stroop effects are reduced with fast responses and are greater as responses slow down unless some inhibition is activated.

Research has identified this pattern of earlier or later interference through the use of the delta plot technique—plotting the effect as a function of response speed [13]. For example, Sharma, Booth, Brown and Huguet [14] showed that the impact of social presence on a Stroop interference task operates by increasing inhibition, as they detected negative slopes in slower responses. To our knowledge, performance on an Ebbinghaus illusion task was not yet analyzed using delta plots, but its dependence of earlier attention mechanisms suggests that no such negative slopes would occur.

Assuming that the performance on Ebbinghaus illusion and Stroop tasks relies upon different attentional mechanisms, one can expect that social presence in the Ebbinghaus task will not replicate the results obtained with social presence in the Stroop task. Since the Ebbinghaus illusion is established in the initial stages of processing, it is less prone to the influence of later inhibition mechanisms. Thus, one should be able to detect the increase in context sensitivity promoted by social presence in this task. In other words, we predict that participants performing the Ebbinghaus illusion task in the presence of others will show increased context sensitivity relatively to those performing it in isolation.

### Current experiment

This experiment explores how social presence modulates individuals' performance on the size perception task associated with the Ebbinghaus illusion. We expect to find evidence of an increased sensitivity to contextual features in participants performing that task in the presence of other participants (co-action) when compared to those performing the same task in an isolated context.

The degree of context sensitivity in this task will be indexed by two variables: the number of correct responses (in which higher accuracy is interpreted as less context sensitivity) and the size of the Point of Subjective Equality (PSE; which is not dependent upon the actual circle size). The PSE represents the point used by individuals to determine whether the target is larger or smaller than the comparison circle, therefore representing the extent to which the response is biased by the context. Both indexes will inform whether individuals in the presence of others perceived the circles differently from those in an isolation condition.

Delta plots will also be computed to assess how attentional mechanisms modulate individuals’ responses. These plots look at the type of responses each participant offered in different time-lags. Following Ridderinkhof’s procedure, individuals’ levels of response accuracy are plotted against their response latencies. Delta plot function's features (e.g., their slopes) reflecting the pattern of context interference are expected to be specifically shaped by social presence. The increase in context sensitivity due to the presence of others, which should be evident in the fastest responses, will promote differences in the levels of accuracy between the two conditions. However, because later inhibition mechanisms are not expected to exert an influence in accuracy, we do not expect social presence to impact the delta curve slopes. More specifically, since those later attentional processes will not interfere with the performance on this task, we predict that delta plots will have the same linear increase with time in both the social presence and isolation conditions.

## Method

### Ethics Statement

This study was reviewed and approved by ISPA-Instituto Universitário Research Ethical Committee. Participants provided their written informed consent to participate in this study. Participants were clearly informed that their collaboration was voluntary and that they could withdraw from the study at any time. The volunteers received a small monetary compensation for their participation.

### Participants and Design

Fifty-seven undergraduates (43 women, *M*_age_ = 22.01; *SD* = 2.24) were randomly distributed into two groups defined by the between-participants factors of a: 2 (social presence: isolation vs. co-action) x 5 (size difference between central circles in the Ebbinghaus figures) mixed design.

Sample size was determined a priori based on relevant previous research data (research reported in this paper that used the same experimental task and analyzed the impact of social presence in a Stroop task).One participant in the isolation condition was excluded because a person entered the room during the experiment and two participants were excluded as they failed to read the instructions and pressed the wrong keys.

### Materials

**Fig 1:**
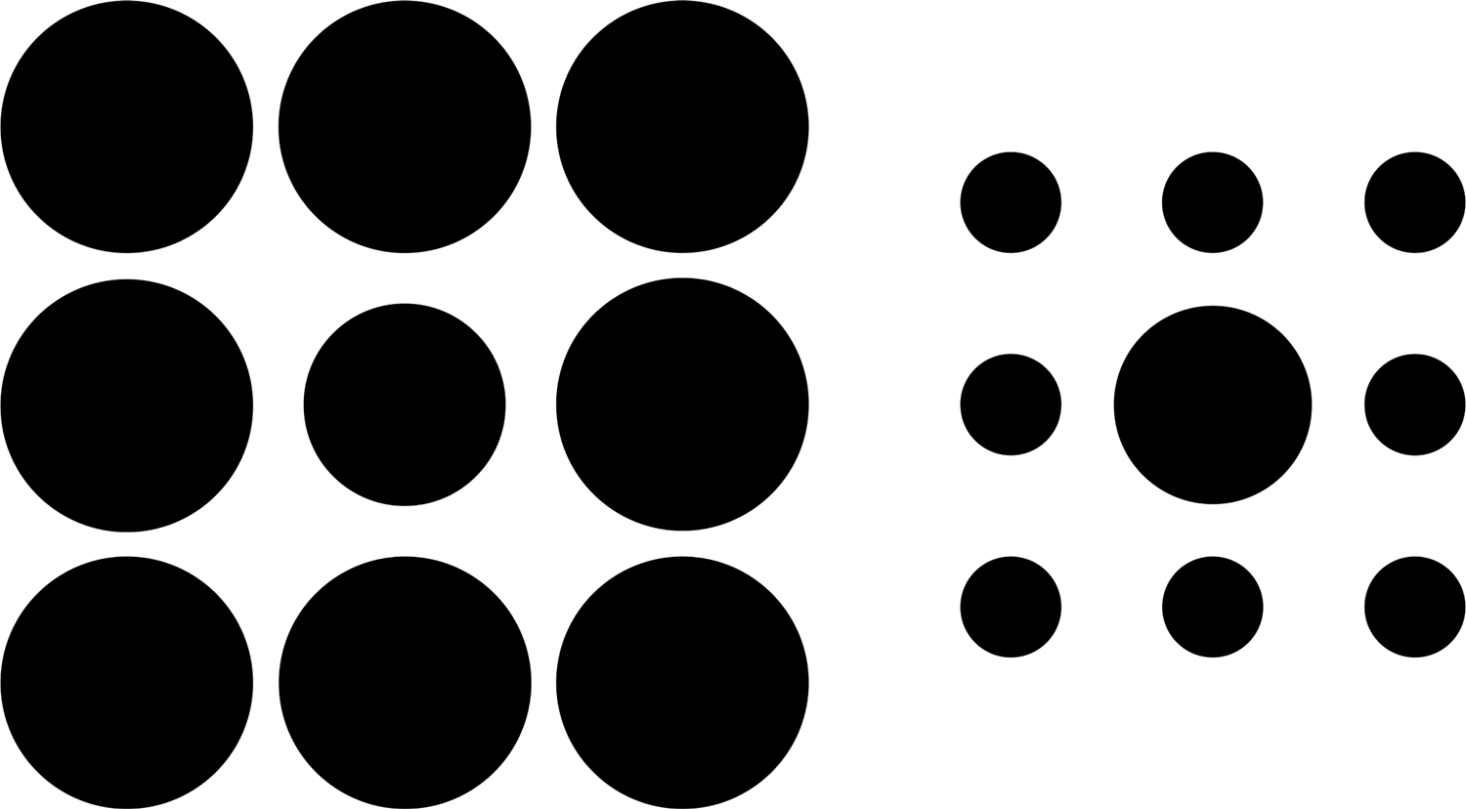
Example of the target stimuli used in this experiment (Ebbinghaus circles). The larger versus smaller surrounding circles makes it difficult to detect the real difference between center circles.

Each trial consisted in the presentation of an image composed of two 3 x 3 arrays of circles, laid out side-by-side (see Fig 1). The center circle of one array had a “standard” size and the central circle of the other array had a different “target” size. The circles that did not occupy the central position of either array were the “surrounding” circles. Each target size was generated by an increase or decrease in the size of the standard circle. The standard circle was 100 pixels in diameter, and the targets were 2, 6, 10, 14, or 18 pixels larger or smaller. Targets with a larger-than-standard circle were always surrounded by even larger circles (125 pixels diameter), and targets with a smaller-than-standard circle were always surrounded by even smaller circles (50 pixels diameter), aiding the illusion.

In some trials, the target was presented at the left side of the screen and the standard at the right side of the screen, and in the other trials, the target was presented at the right and the standard at the left of the screen. Moreover, in some trials, the target was larger than the standard and in the other trials the target was smaller than the standard, by one of the five size differences (i.e., the 2, 6, 10, 14, or 18 pixel difference). The crossing of these features (i.e., larger target vs. smaller target X target at the left vs. target at the right) produced 20 different kinds of trials. Each one of these kinds of trials was presented four times in such a way that participants evaluated a total of 80 incongruent target trials (i.e., trials in which the context induces an incorrect response; e.g., larger surrounding circles induce perceptions of large targets as being smaller circles). But because in these trials the smaller of the two center circles was always surrounded by smaller circles and the larger by larger circles, individuals could use a simple strategy of providing a response by attending to the array, which would coincide with the correct answer. To avoid this behavior, filler trials with 98 and 102 pixels circles, surrounded by circles of 125 pixels and 50 pixels, respectively, were presented either on the right or the left of the screen.

### Procedure

After reading and signing the informed consent form, the participants were invited to go to the laboratory at a specific time. Participants arrived at the lab either at the same time as other colleagues or alone and were welcomed by an experimenter that explained that all instructions for participation would be given on the computer screen after they initiated the study. In the co-action condition, participants were seated side by side with other participants (tables of 90 cm with a divider that prevented them from seeing one another's computer screens). Thus, in this co-action condition, participants were aware of other participants in the experiment. In the isolation condition, participants were by themselves and the experimenter left the room after giving them the general initial instructions. All participants were instructed to return to the front desk to receive the agreed payment after task completion.

The study was run using the E-Prime 2.0 software. The instructions stated that the participant’s task was to quickly decide which of two figures contained a bigger center circle by using the left and right arrow keys of the keyboard. Trials were presented in a random order.

## Results

The accuracy on trials with larger targets surrounded by smaller shapes was 100%, suggesting that any errors in the critical trials reveal the influence of the context. An index of the context sensitivity effect was obtained by calculating the total number of 16 possible correct responses (four repetitions of the four trial types: larger vs. small x left vs. right) for each of the five size differences combined (excluding congruent trials). This index increased as context sensitivity decreased.

**Fig 2:**
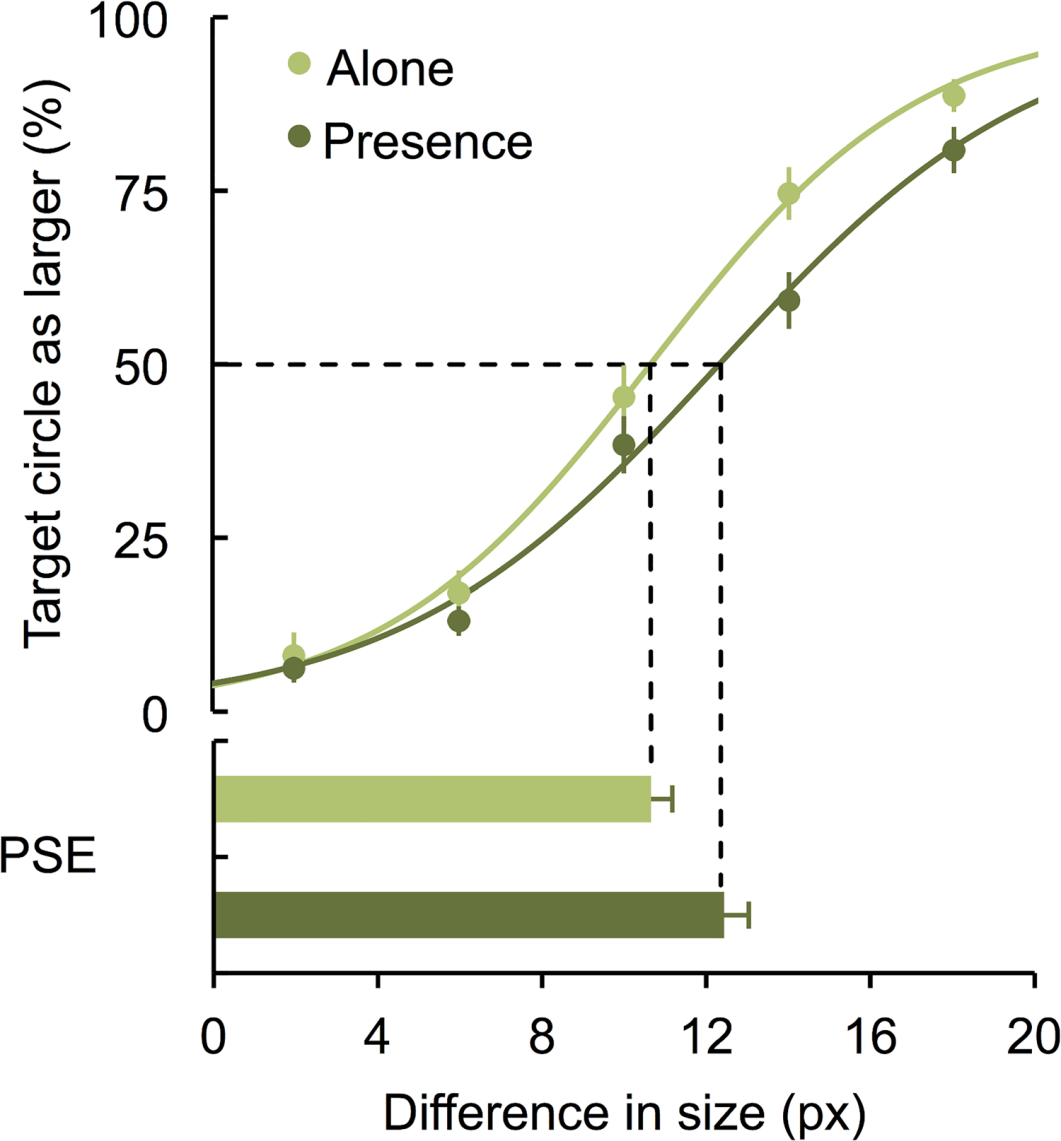
Accuracy of participants in isolation and co-action conditions as a function of size differences for the conditions in which the larger center circle was surrounded by even larger circles. Point of subjective equality (PSE) for each group.

Across all conditions participants showed the expected evidence of context sensitivity (mean proportion of correct responses = 42.25%; *SD* = 13.42%). We further compared the levels of accuracy in an 2(co-action vs. isolation context) x 5 (size difference) mixed design ANOVA. Because the context influence is more likely to occur in more ambiguous trials (i.e., when the size of the target circle is closer to the size of the standard circle), we expected a main effect of the size difference factor reflecting a linear trend. This significant trend, *F*(4, 216) = 292.30, *p* < .001, η^2^_partial_ = 0.84, is illustrated in Fig 2, which shows lower accuracy levels for small differences (2 pixel difference from standard) and higher accuracy for bigger differences (18 pixel difference from standard). The predicted social presence effect was also marginally significant, *F*(1, 55) = 3.34, *p* = .073, η^2^_partial_ = 0.06, suggesting that participants in co-action (*M* = 46.56%, *SD* = 10.49%) were more context sensitive than those who performed the task alone (*M* = 39.86%, *SD* = 14.38%). A two-way interaction, *F*(4, 216) = 2.54, *p* = .040; η^2^_partial_ = 0.05, suggested that this increased accuracy of participants in the isolation condition did not occur when the task was more difficult (smaller differences, *t*<1) but rather when the size difference was more noticeable, *t*(54) = 2.34, *p* = .023, *d* = 0.64.

To understand whether participants in isolation differed from those in co-action in their subjective size perception, we determined the PSE (see Fig 2) for each participant by fitting a logistic function to the data (mean R^2^ = 0.94, *SD* = 0.27) and determining its 50% of accuracy point (i.e., the point of subjective equality—PSE). Participants in each experimental condition differed significantly in their PSE values, *t*(54) = 2.03, *p* = .046, *d* = 0.55. Those in co-action condition perceived the difference between circles as bigger (*M* = 13.17, *SD* = 5.11) than those in the isolation condition (*M* = 10.74, *SD* = 1.92). This pattern is exactly what we would expect if the presence of others augments context sensitivity.

### Time Course Analysis

We further compared the two experimental conditions in their response time features and delta plots.

Delta plots were calculated for each participant. To do so, first we ranked the reaction times (RT) of all responses (correct and incorrect) and divided into four equal-size speed bins (quartiles). Mean RT for correct and incorrect responses and mean accuracy level were subsequently determined for each quartile. The equivalence of these bins in each experimental condition was analyzed, having the correct and incorrect responses RTs of each bin as two within factors in the mixed ANOVA that contrasted the two experimental conditions. The tautological main effect found for bins, *F*(3, 165) = 82.64, *p* < .001, did not interact either with the social presence factor (*F* < 1) or with accuracy (*F* < 1), suggesting that the RT bins were equivalent in isolated and co-action participants and in correct and incorrect responses.

**Fig 3:**
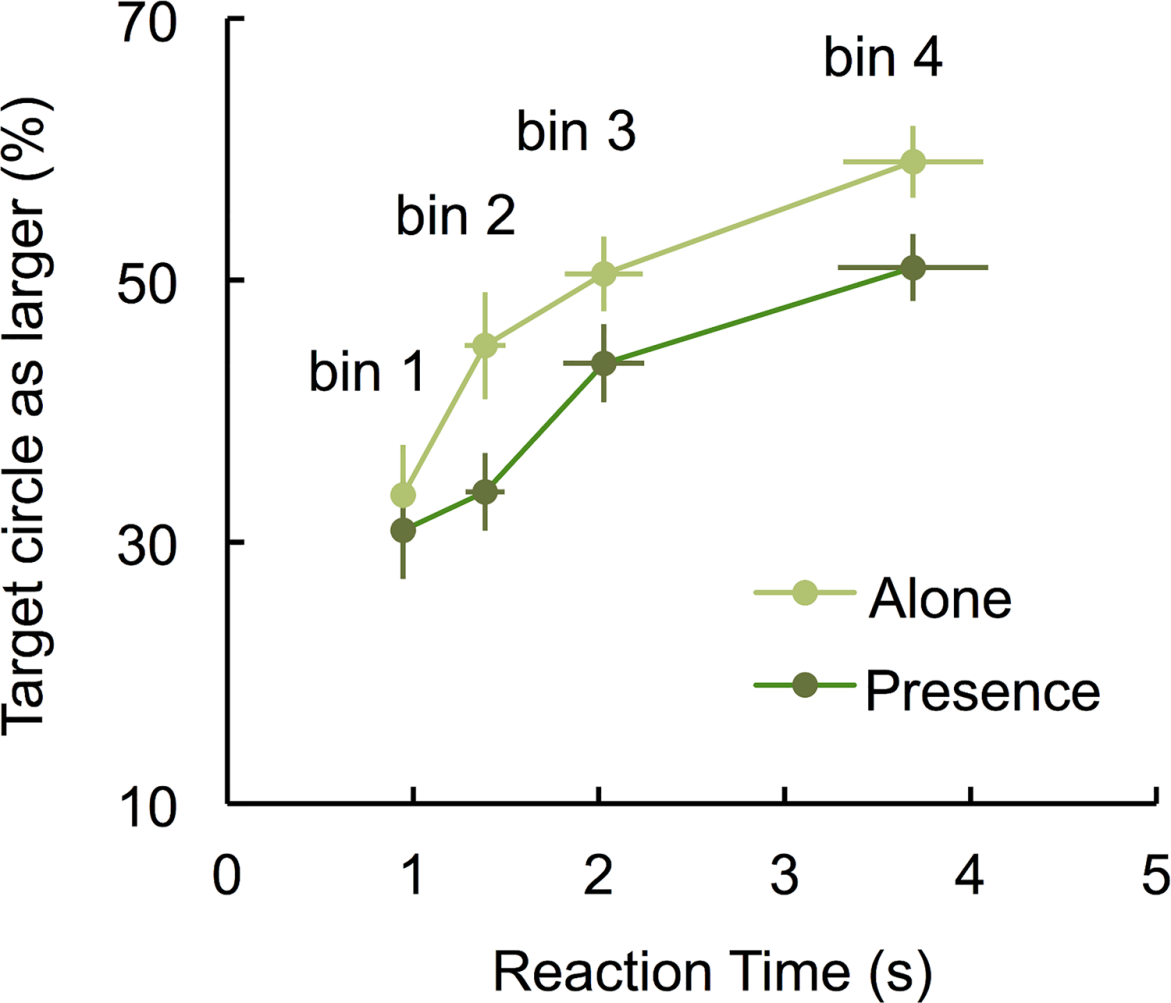
Accuracy of participants in isolation and co-action conditions as a function of the quartiles of reaction times (delta plots) when the larger center circle was surrounded by even larger circles.

Delta plots (see Fig 3) were then created for each experimental condition by plotting the proportion of correct responses (accuracy) as a function of response speed (i.e., per bin). The general delta plot function defined a positive linear trend, *F*(3, 162) = 28.48, *p* < .001, η^2^_partial_ = 0.34, with no quadratic component (*F*<1). Delta plots showed that the interference occurred immediately in initial processing of stimuli and was reduced when individuals took more time to perceive the stimuli (a pattern that opposes the one observed in the interference scores of Stroop-like tasks, in which interference needs time to be implemented). The same linear trend occurred in both experimental conditions (interaction: *F* < 1) suggesting that the increase in performance with time was similar in both conditions. A careful analysis of Fig 3 suggests, however, that individuals in the isolation condition were quicker to disentangle context effects than individuals in the co-action condition. The performance of those in the isolation condition improved significantly from bin 1 to 2, *t*(54) = 3.07, *p* = .003, *d =* 0.84, whereas performance in the co-action condition did not, *t*(54) = 1.07; *p* = .287.

In order to better contrast experimental conditions regarding the levels of context interference in different response times, we followed Ridderinkhof [12] and computed each individual’s partial curve slope (slope segments connecting the data points of quartiles 1 and 2, quartiles 2 and 3, and quartiles 3 and 4). We calculated the difference between the two delta points relative to the time difference between bins for that specific individual [q2-q1/(RT2-RT1)]. Because of the interdependency of these data, we analyzed the effects through the comparison of their 95% confidence intervals [15] (see Table 1). As previously suggested, isolated and co-action conditions differed in the extent that performance in the isolation condition started to improve earlier (in slope 1) than in the co-action condition (only in slope 2, since slope 1 is not significantly different from zero). Congruently with our predictions, co-action participants were more prone to context influences. Importantly, this analysis also suggests that in this Ebbinghaus illusion task the presence of other participants did not lead to a more efficient control of the context interference in size judgments. The type of interference that occurs in the Ebbinghaus illusion task clearly differs from the type of interference observed in a Stroop task, which promotes differences between isolated and co-action conditions in the last slope. Here, the confidence intervals completely overlapped, suggesting no such difference. An additional piece of information revealed by this analysis was that the curve slopes were all close to zero, suggesting that time quickly became irrelevant to help individuals oppose context influences.

**Table 1:** Mean Slopes and 95% CI of each Social Presence Condition

		Slope 1^a^	Slope 2 ^a^	Slope 3 ^a^
Isolation	Mean	.267	.118	.055
	95% CI	[.032; .471]	[-.107; .346]	[.001; .111]
Co-Action	Mean	.068	.257	.063
	95% CI	[-.099; .235]	[.086; .429]	[.040; .123]

ª Partial curve slopes, S1 = slope segments connecting the data points of quartiles 1 and 2; S2 = slope segments connecting the data points of quartiles 2 and 3; S3 = slope segments connecting the data points of quartiles 3 and 4.

## Discussion

The results of our experiment showed that participants in the presence of others perform worse at an Ebbinghaus illusion task than participants in isolation. Both the number of correct responses and the PSE index, reflecting context influences, suggest that participants in a social presence condition were more sensitive to the features of the context.

The analysis of the delta plots allows us to understand that that time does not favor the effect in the Ebbinghaus illusion task. Time is only relevant in the process of preventing the illusion from occurring (in opposition to what happens in a Stroop task). Additionally, the delta plots analysis showed no evidence of the impact of social presence in enhancing control over the context influence, like the one previously observed in a Stroop task. The general pattern of data seems thus to corroborate the assumption that in the Ebbinghaus illusion task, interference is quickly established (immediately influencing the percept apprehension), and that control mechanisms, in order to be efficient, need to occur in an earlier phase of processing. Participants either perceived the center circle ignoring the context, or perceived it incorporating the context into the percept, with the latter occurring more frequently in participants performing the task in co-action. Additionally, co-action participants seemed to have more difficulty ignoring context influences than those in isolation (who showed a significant increase in performance even when providing quick responses, represented by slope 1). For those in co-action, only more delayed responses ignored the context.

These results corroborate our initial idea that the Ebbinghaus task is better able to detect social presence effects on local/global perception (i.e., similar to what is observed in the framed-line test) than social presence effects on executive control function.

Although this experiment was not designed to compare between various explanations of social facilitation, it offers some relevant insights. The hypothesis that social presence effects are related to an increase in negative arousal (e.g., mere presence, evaluation apprehension, perceived threat) would predict that participants would process the stimuli in a more detailed way, reducing the sensibility to holistic features of the perception [16, 17]. Our results contradict this prediction. The hypothesis that social presence leads individuals to focus on relevant stimuli and less on irrelevant stimuli [18] would suggest that participants in the presence of others, and thus with increased attention to relevant stimuli, would have reduced illusions of size. Our results do not support this prediction either. Additionally, these data bring some insight to the approach suggested by Zajonc [19, 20], who hypothesized that social presence increases reliance on well-learned responses, which could lead to better or worse performance depending on the difficulty of the task. In our experiment, when we looked at the results of easy (i.e., the standard and target circles had a big size difference) and difficult (i.e., the standard and target circles had a small size difference) trials, we did not find the expected moderation. Accordingly, the participants in the social presence condition, across all levels of task difficulty, performed worse than those in isolation, which challenges this perspective. Blascovich, Mendes, Hunter and Salomon [21] suggest that social presence simply enhances task-engagement. According to these authors’ view, our experimental situation would promote challenge (and not threat) because individuals have the cognitive resources to address the task. A challenged state is associated with superficial holistic processing [22] and could thus account for the observed effects. However, this hypothesis, which is definitely worthy of further testing, is less parsimonious than an assumption of increased context sensitivity.

Future research should also focus on the relationship of this social presence effect with context sensitivity differences such as: a) gender [6], as women have been shown to be more social than men; b) culture [3], as Americans feel more isolated from a social context than Japanese; and c) pathologies such as schizophrenia (for a review, see [23]), in which decreased sensitivity to the context may be related with social isolation.

Since the inception of social psychology, we have constantly generated and accumulated knowledge about the impact of others on our behavior, but we are still searching for a clearer answer to the question of why social presence modulates our thoughts and behavior. The more we know, the more we are able to ask about this phenomenon.
